# Clinical study on improving postoperative symptoms of cervical spondylotic myelopathy by Qishe pill

**DOI:** 10.1097/MD.0000000000021994

**Published:** 2020-09-04

**Authors:** Jinhai Xu, Xiaoning Zhou, Chen Xu, Chongqing Xu, Xing Ding, Kun Jin, Ming Yan, Junming Ma, Xuequn Wu, Jie Ye, Wen Mo, Wen Yuan

**Affiliations:** a Department of Orthopaedics, LongHua Hospital, Shanghai University of Traditional Chinese Medicine; b Department of Orthopaedics, Changzheng Hospital, Second Military Medical University, Shanghai, China.

**Keywords:** Cervical spondylotic myelopathy, Postoperative residual symptoms, Qishe pill, Randomized controlled trial

## Abstract

**Background:**

Cervical spondylotic myelopathy (CSM) is the most serious type of cervical spondylosis, which is often treated surgically in patients with progressive neurological symptoms following ineffective conservative treatment. However, some patients have residual symptoms such as neck pain, stiffness, and C5 nerve palsy after surgery. The Qishe pill can effectively relieve the symptoms of neck pain and numbness, but there is no evidence showing the efficacy and safety of the Qishe pill in treating symptoms after spinal cord surgery.

**Methods/design:**

A multicenter, randomized controlled clinical trial will be conducted to evaluate the efficacy and safety of the Qishe Pill. A total of 330 patients with CSM who receive surgical treatment will be randomly divided into 2 groups, treated for 12 weeks and with a 1-year follow-up. The primary outcome will be Japanese Orthopaedic Association score from the baseline to 4 weeks, 12 weeks, 24 weeks, and 48 weeks after surgery. Secondary outcomes will include Visual Analogue Scale score, Neck Disability Index, and imaging indicators (including magnetic resonance imaging and X-ray). Additionally, adverse reactions will be observed and recorded as safety indicators.

**Discussion:**

Although the Qishe pill can effectively improve the discomfort of the neck and upper limbs in clinical applications, there is a lack of clinical research on postoperative patients. This study will investigate the efficacy and safety of the Qishe pill in treating postoperative symptoms of CSM.

**Trial registration:**

Clinical Trials.gov ID: ChiCTR1900028173. Registered on 17 December 2019.

## 1 Introduction

Cervical spondylotic myelopathy (CSM) often presents with symptoms that are related to dysfunction of the spinal cord, caused by the degeneration of the cervical vertebrae (such as intervertebral disc, ligaments, osteophytes) and related secondary changes (excessive activity of the cervical vertebrae and cervical instability).^[1,2]^ By age 50, magnetic resonance imaging (MRI) showed that as many as 31.6% of the population had spinal cord compression, rising to 66.8% after age 80.^[3]^ Progressive CSM can cause a range of symptoms such as limb numbness, walking instability, and bundle sensation, and 20% to 60% of spinal or neurological symptoms can worsen without surgery.^[4,5]^ Therefore, an increasing number of patients are receiving surgical treatment because it can relieve nerve compression, stop the progression of nerve damage, and improve function and quality of life. Although surgery is usually safe and effective, complications occur in 11% to 38% of patients. Fehlings et al^[6]^ found that overall perioperative and delayed complication rates were 15.6% and 4.4%, respectively. Most complications are temporary and non-neurological. No invasive intervention or prolonged hospital stay is required. However, some patients have intractable neck pain, limb numbness, and even muscle weakness after surgery.^[7,8]^

The Qishe pill is a Chinese medicine consisting of processed Radix Astragali, Muscone, Szechuan Lovage Rhizome, Radix Stephaniae Tetrandrae, Ovientvine, and Calculus Bovis Artifactus, and has the function of regulating qi and promoting blood circulation, regulating collaterals, and relieving pain.^[9,10]^ Related studies showed that it can effectively relieve cervical pain and numbness, and improve quality of life in patients with CSM.^[11,12]^ It has been found in clinical practice that the Qishe pill can improve postoperative symptoms of CSM to some extent, but there is still a lack of evidence to prove this. Therefore, we designed a multicenter, randomized, clinically controlled trial to demonstrate its efficacy and safety.

## 2 Materials and methods

### 2.1 Study design

**Figure 1:**
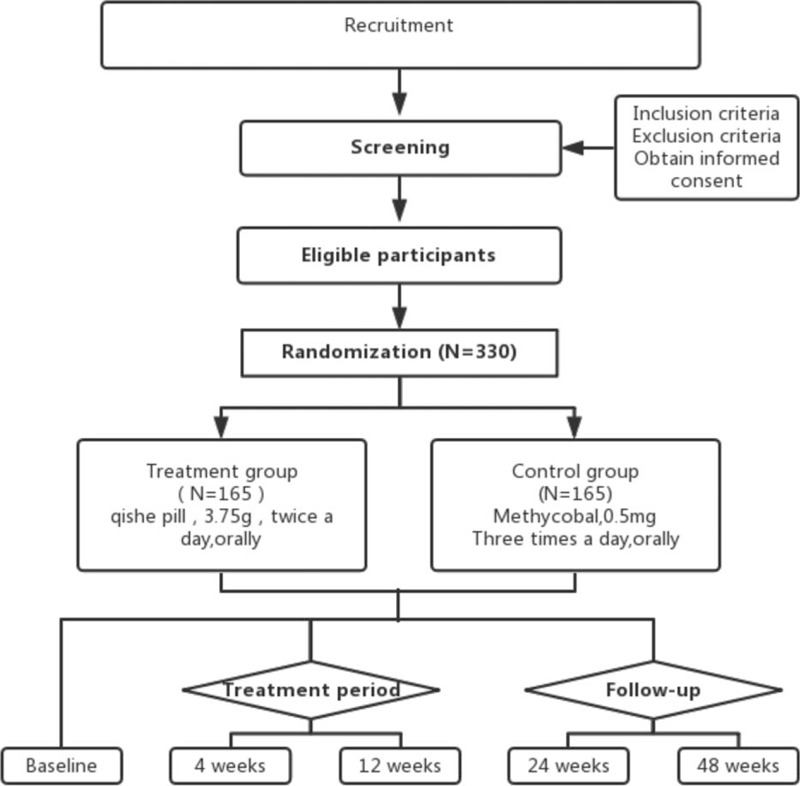
Project overview.

This is a multicenter, randomized clinical trial with 2 parallel arms (Fig. 1). We will recruit 330 patients from 2 medical institutions in Shanghai, China: Longhua Hospital Affiliated with Shanghai University of Traditional Chinese Medicine, and Long March Hospital Affiliated with Second Military Medical University. All patients will be randomly assigned to either an experimental group or a control group. Patients in the experimental group will be required to take the 3.75-g Qishe pill twice daily after breakfast and dinner, and patients in the control group will take 1 Methycobal tablet 3 times daily. All drugs will be administered orally for 12 weeks.

### 2.2 Ethical issues

The trial will be conducted in accordance with the Declaration of Helsinki and Ethical Guidelines for Clinical Research, and the trial protocol has been approved by the Research Ethical Committee of Longhua Hospital Affiliated with Shanghai University of Traditional Chinese Medicine, Shanghai, China, (approval number: 2019LCSY081).

All patients will be informed of the protocol, the purpose of the trial, and the rights, obligations, and risks involved with participation in the trial. Only patients who fully understand and sign the informed consent form will participate in the trial. In addition, the personal information about potential and enrolled subjects will not be shared or maintained if unnecessary.

### 2.3 Study participants

We will recruit patients using posters and from outpatient and inpatient departments from 2 hospitals (LongHua Hospital Affiliated with Shanghai University of Traditional Chinese Medicine and Long March Hospital Affiliated with Second Military Medical University). The planned recruitment period is 12 months.

### 2.4 Sample size calculation

On the basis of the comparison treatment, we calculated the sample size of the 2 groups (Qishe pill versus Methycobal), which will be randomly distributed in an equal manner. Since Japanese Orthopaedic Association (JOA) scores can include sensory and motor function of the limbs and bladder, we chose JOA scores to calculate the sample size with an estimated correlation coefficient of the measurements of ρ=0.8 and with an alpha = 0.05. The JOA score was 13.68 ± 1.83 at the 6-month follow-up after oral administration of Methycobal, and 15.65 ± 1.61 at the 6-month follow-up after oral administration of traditional Chinese medicine for promoting blood circulation and removing blood stasis, so the calculated sample size of each group is 132. Considering a 20% loss to follow-up, the total sample size needed to detect this difference at a 5% level of significance with a power of 90% is 330 patients.

### 2.5 Criteria

#### 2.5.1 Inclusion criteria

Participants will be included if they fulfill the following conditions:

(1) Satisfy the diagnostic criteria of CSM, and have definite spinal cord compression on MRI of cervical vertebrae^[13]^
(2) Aged 18 to 80 years
(3) Will be receiving surgical treatment (including anterior and posterior)
(4) Sign informed consent

#### 2.5.2 Exclusion criteria

Patients with any of the following conditions will be excluded:

(1) Those who are pregnant, nursing or preparing for pregnancy
(2) Those with diseases of the liver, kidney, hematopoietic system, or endocrine system, or other serious primary and mental diseases
(3) Those with other spinal diseases, tumors, or cerebral infarction that affect limb sensation and function
(4) Those who are participating in clinical studies of other drugs

#### 2.5.3 Interventions

All patients will receive routine anti-inflammatory and analgesic treatment after surgery.

Experimental group: Patients will be given the Qishe pill (orally twice a day, 1 package (3.75 g) at a time. Control group: Methycobal will be taken orally 3 times a day, 1 tablet at a time. The course of treatment will be 12 weeks.

### 2.6 Randomization and allocation

We will randomize all included patients using a table of random numbers. The 288 patients will be randomly divided into the experimental group and the control group in a 1:1 ratio according to the treatment order.

### 2.7 Outcomes

#### 2.7.1 Primary outcomes

The primary outcomes include the items:

JOA scores assessed the patients’ cervical spine function based on the sensation and movement of the limbs, sensation of the chest and abdomen, and bladder function. In addition, the improvement rate of JOA will be compared.^[14]^ 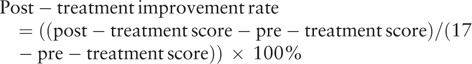


#### 2.7.2 Secondary outcomes

**Figure 2:**
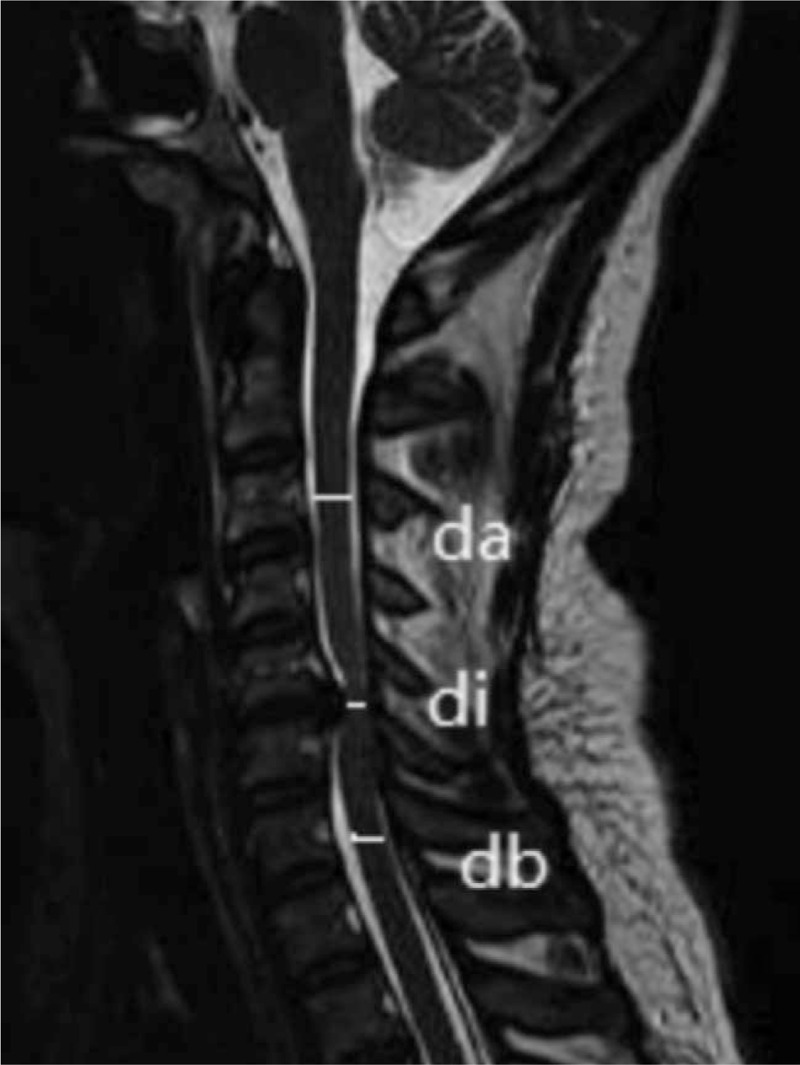
Measurement of percentage of median sagittal spinal cord compression in T2WI. T2WI = T2 weighted image.


The secondary outcomes include the following 3 items: 

(1) Visual Analog Score (VAS): VAS will be used to assess the patients’ pain level: Some patients still have pain after surgery, and VAS will be used to evaluate the improvement of pain after oral Qishe pill/Methycobal.^[15]^
(2) Neck Disability Index: Neck Disability Index will be used to assess the patients’ ability to live and work; the higher the value, the more severe the dysfunction.^[16]^
(3) Increased Signal Intensity: CSM usually has increased signal intensity on MRI, and changes in spinal cord signals will be observed before and after treatment.^[17,18]^
(4) Maximum Spinal Cord Compression (Fig. 2): Spinal sagittal diameters (DI) of the most severely compressed segments and the spinal sagittal diameters (DA, DB) of the uncompressed segments at the upper and lower segments will be measured at the median sagittal position of T2 weighted image (T2WI). The formula is

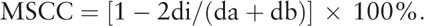

**Figure 3:**
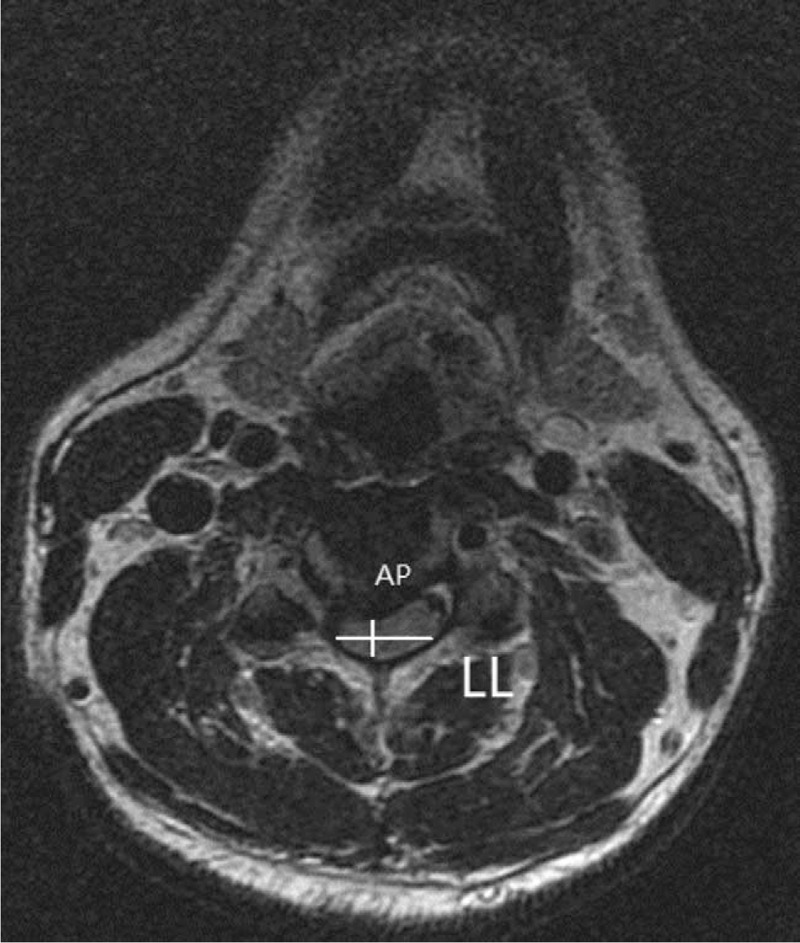
Measurement methods of anterior and posterior AP and lateral LL of T2WI compressed spinal cord. AP = Anteroposterior, LLT2WI = T2 weighted image.

**Figure 4:**
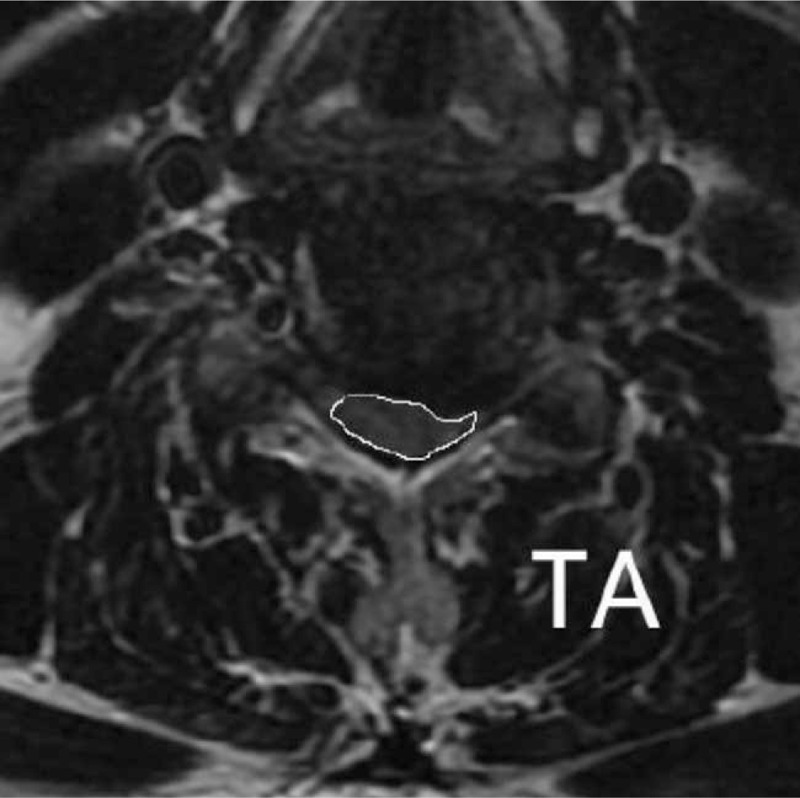
Measurement of the cross-sectional area of the spinal cord at the horizontal axis of T2WI. TA = transverse area, T2WI = T2 weighted image.

(1) Compression Ratio (Fig. 3): compression ratio is the ratio of anteroposterior (AP) to laterlateral (LL). AP is the anterior and posterior diameter of the spinal cord at the level of the most severe spinal cord compression on the horizontal axis of T2WI, while LL is the left and right diameter.
(2) Transverse Area (Fig. 4): The cross-sectional area of the most severely compressed spinal cord on T2WI will be measured.

### 2.8 Safety assessments

Qishe pill has been widely used in clinical practice, and has been found to be safe in clinical trials. Patients will be closely monitored for adverse reactions during this trial. In addition, laboratory tests will be performed at the beginning and end of the trial, including blood, urine, fecal, kidney, and liver function tests. If adverse events are reported, we will provide appropriate treatment to participants immediately.

### 2.9 Participant timeline

The study will be followed for 96 weeks, from December 30, 2019 to December 30, 2021. Recruitment will start in December 30, 2019 and December 30, 2020. The recruitment process is shown in Figure 1, and the schedule is shown in Table 1.

**Table 1:**
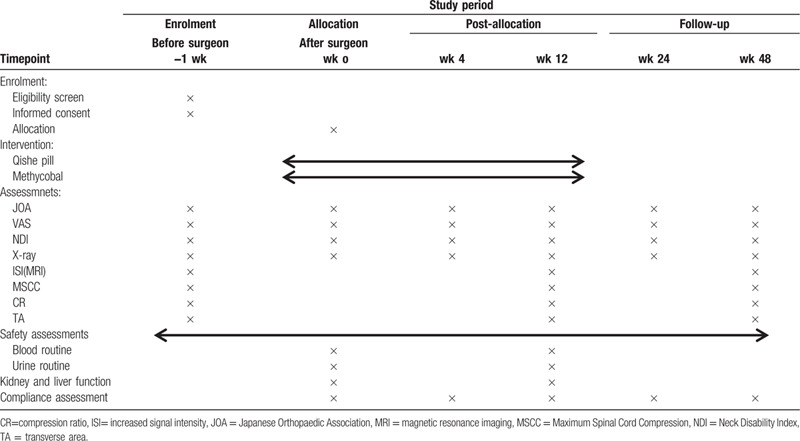
Schedule of enrollment and assessments.

### 2.10 Data collection and monitoring

The duration of the intervention will be 12 weeks, and follow-up will be 36 weeks. Study visits will take place at baseline and at 4, 12, 24, and 48 weeks. Every patient will be asked to visit within 3 days of the given time point. Longhua Hospital affiliated with Shanghai University of Traditional Chinese Medicine (http://www.longhua.net/ywsy/gzlc/287.jhtml) will be responsible for quality control and training for investigators.

Epidata (version 3.0) will be used to restrict data values. Two trained investigators will enter and compare the data independently to rule out the differences.

### 2.11 Quality control

All staff in the clinical study will be relatively fixed and receive unified training before the clinical trial starts, so that all investigators have a good understanding of the clinical trial protocol. All investigators will be familiar with the recording method of the case observation table and judgment standard, and will be able to teach the recording method to patients. All of the cases will be filled according to the design requirements of the “Case Record Form”. Drop-outs and withdrawals (and the reasons) from the study will be fully documented. and 2 inspectors will be appointed to supervise and inspect the research process.

### 2.12 Statistical analysis

Statistical analysis includes statistical description and statistical inference. The statistical description of quantitative index includes mean, standard deviation, median, minimum, and maximum. The qualitative index is described by frequency table and composition ratio. Statistical inference includes interval estimation and hypothesis testing.

All statistical analysis will be conducted by Statistical Packages of Social Sciences software (version 22.0). By the bilateral test, *P* < .05 will be considered to be statistically significant. The mean ± standard deviation will be used for the description of continuous variables. Continuous variables following the normal distribution will be analyzed by Student *t*-test. The non-normally distributed data will be tested using the rank sum test. The non-grade data in the counting data will be tested with the 2 test or the non-parametric test. The grade data will be checked by Ridit.

## 3 Discussion

Patients with CSM have a high rate of surgery, and some patients have obvious residual symptoms.^[19]^ In the absence of established guidelines, treatment should center around relief of patient symptoms. Treatment modalities include bed rest, medications, physical exercise, thermal therapy, and others.^[20,21]^ Therefore, it is important to explore new treatment options to alleviate postoperative symptoms and improve the quality of life of patients. To the best of our knowledge, this is the first randomized controlled clinical trial to investigate the efficacy of the Qishe pill in the treatment of postoperative residual symptoms of CSM.

The Qishe pill is a combination of eight herbal ingredients and has been widely used in the clinical setting, and can significantly reduce the stiffness and numbness of the neck and shoulder. It is recommended for treating a variety of ailments, including neck pain, radiating pain, limb stiffness and numbness, weakness, fatigue, and other symptoms. Related studies have proven that astragalus and chuanxiong have the effects of nourishing qi, generating blood, and improving circulation.^[22,23]^ However, there remains a lack of evidence for the residual neck and shoulder pain and numbness of CSM. Therefore, this clinical study will be conducted to explore the efficacy and safety of the Qishe pill to provide a new therapeutic scheme for clinical improvement of postoperative residual symptoms of CSM.

## Acknowledgments

We would like to thank Longhua Hospital Clinical Evaluation Center, Shanghai University of Traditional Chinese Medicine.

## Author contributions

Jinhai Xu, Xiaoning Zhou and Chen Xu are co-first authors of this manuscript. Contributing equally to the design, conduct of the trials, and drafting of the manuscript. All authors participated in the design of the study and performed the trial. Jie Ye, Wen Mo and Wen Yuan are the co-corresponding authors of this manuscript, contributing equally to the supervision and coordination of the clinical trial. All authors read and approved the final manuscript.
